# Home Smoking Bans and Urinary NNAL Levels to Measure Tobacco Smoke Exposure in Chinese American Household Pairs

**DOI:** 10.3390/ijerph18147682

**Published:** 2021-07-20

**Authors:** Emiley Chang, Melanie Dove, Anne Saw, Janice Y. Tsoh, Lei-Chun Fung, Elisa K. Tong

**Affiliations:** 1 Department of Medicine, Harbor-UCLA Medical Center, 1000 W. Carson St, Torrance, CA 90502, USA; ey3chang@ucla.edu; 2 David Geffen School of Medicine, University of California—Los Angeles, 10833 Le Conte Ave, Los Angeles, CA 90095, USA; 3 The Lundquist Institute, 1124 W Carson St, Torrance, CA 90502, USA; 4 Department of Public Health Sciences, University of California—Davis, One Shields Ave, Medical Sciences 1-C, Davis, CA 95616, USA; mdove@ucdavis.edu; 5 Department of Psychology, DePaul College of Science and Health, 2219 North Kenmore Ave, Chicago, IL 60614, USA; asaw@depaul.edu; 6 Department of Psychiatry, University of California—San Francisco, 401 Parnassus Ave, San Francisco, CA 94143, USA; janice.tsoh@ucsf.edu; 7 Chinatown Public Health Center, San Francisco Department of Public Health, 1490 Mason St, San Francisco, CA 94133, USA; lei-chun.fung@sfdph.org; 8 Department of Medicine, UC Davis Medical Center, 2315 Stockton Blvd, Sacramento, CA 95817, USA

**Keywords:** tobacco smoke exposure, tobacco-specific nitrosamine, NNAL, biomarker, household pairs, home smoking ban, Chinese American, immigrant health

## Abstract

Home smoking bans can reduce tobacco smoke exposure, but little is known about the impact for Chinese American household pairs. In this study of 202 household pairs with low acculturation, 53.9% reported a home smoking ban, 31.7% had inconsistent reports, and 14.4% reported no ban. With decreasing home smoking ban enforcement, more nonsmokers had tobacco smoke exposure (66.1%–86.2%) as measured by the tobacco-specific nitrosamine biomarker urine NNAL (4-(methylnitrosamino)-1-(3-pyridyl)-1-butanol). Despite reported bans, about one-quarter of nonsmokers still reported tobacco smoke exposure at home (23.6%–30%) within the past 2 months and three-quarters reported outdoor exposure. In adjusted regression analyses of geometric mean NNAL ratios, nonsmokers in households with no ban had over two times higher levels than nonsmokers in households with a ban: adjusted log NNAL ratio = 2.70 (95% CI 1.21, 6.03). Higher smoker NNAL level and nonsmoker English fluency were also significantly associated with nonsmoker NNAL levels. Nonsmoker levels in households with an inconsistent ban were not significantly different compared to those with a ban. Although home smoking bans were generally associated with lower NNAL levels, tobacco smoke exposure in this immigrant population with low English proficiency was higher than that of the general population. From a health equity standpoint, there is a need for broader implementation and enforcement of comprehensive smoke-free policies.

## 1. Introduction

As homes are an important source of tobacco smoke exposure, home smoking bans are a powerful method to encourage smokers to stop smoking and protect the health of nonsmokers [1,2,3]. The Surgeon General has concluded there is no safe level of tobacco smoke exposure, which increases the risk of cancer, respiratory, and cardiovascular diseases [4,5]. However, only half of U.S. households with at least one adult smoker reported having a home smoking ban in the 2014–2015 Tobacco Use Supplement to the Current Population Survey [6].

Most population-based assessments of tobacco smoke exposure rely on questionnaires due to the relative ease of administration compared to biomarker collection or environmental monitoring [7]. Among biomarker (cotinine) studies examining children’s tobacco smoke exposure, studies from Australia, the U.K., and Italy [8,9,10] showed that home smoking bans in households with a current smoker were associated with lower tobacco smoke exposure; however, a U.S. study of North Carolina black mother–child dyads did not find a difference [11]. Of note, these studies relied on a single reporter for home smoking ban status. However, individuals within the same household may have different views or understandings about their home smoking ban, which may reflect lax enforcement or partial restrictions and result in higher levels of exposure among nonsmokers [12,13,14]. Indeed, in a 2011 study of 388 multiethnic households with at least one smoker, 19% of parent–child pairs had discordant reports of home smoking ban status [15]. Tobacco smoke exposure, as measured by cotinine, was lowest when the pairs agreed there was a home smoking ban, intermediate when the pairs were inconsistent, and highest when the pairs agreed there was no home smoking ban. Combining parent and child reports explained more variance in child cotinine levels compared to either type of report alone.

Tobacco smoke exposure in the home may be particularly important for Asian American immigrants, whose countries of origin have a high male smoking prevalence but low female smoking prevalence. In a population-based study of Chinese Americans in New York City [14], only one-third of current smokers reported a home smoking ban compared with 79% of former smokers and 71% of never smokers. In a population-based study of Chinese and Korean American women in California, 58% reported having a smoke-free policy in their home. However, there were differences in enforcement of this policy. Women with less than a high school education were more likely to report someone smoking at home compared with higher educated women, regardless of the home smoking ban [16].

The purpose of this study is to compare home smoking ban status, reported by Chinese American smoker and nonsmoker household pairs in the San Francisco area and tobacco smoke exposure levels in nonsmokers using a tobacco-specific nitrosamine biomarker NNAL (4-(methylnitrosamino)-1-(3-pyridyl)-1-butanol). We hypothesized that household pairs that agreed there was a home smoking ban would have the lowest levels of nonsmoker NNAL levels, followed by household pairs with inconsistent reports and no home smoking bans.

## 2. Methods

We conducted a cross-sectional analysis of baseline surveys independently completed by 202 pairs of male Chinese American smokers and female household nonsmokers enrolled in a randomized controlled trial between December 2012 and April 2015 [17]. The study was approved by the Institutional Review Board of the University of California, Davis. Informed consent was obtained from all subjects involved in the study. The study was a community—academic collaboration between Chinatown Public Health Center (CPHC), University of California San Francisco, and University of California, Davis. CPHC recruited smoker-nonsmoker household pairs from the San Francisco area through media outreach, community organizations, health care facilities, and health fairs. Pairs were eligible to participate if they were 18 or older, self-identified as Chinese American, and were composed of a male smoker and household nonsmoker. Surveys were translated into Chinese by health education staff and included adapted items from population-based tobacco surveys.

### 2.1. Tobacco-Specific Nitrosamine Biomarker of Tobacco Smoke Exposure

All participants submitted daytime urine samples to analyze levels of NNAL (4-(methylnitrosamino)-1-(3-pyridyl)-1-butanol), a tobacco-specific carcinogen that is a biomarker of tobacco smoke exposure over the past 1–2 months [18]. The long elimination half-life of NNAL improves cumulative detection of intermittent tobacco smoke exposure compared to cotinine, which only reflects the past few days of exposure [19,20] and has varying racial/ethnic cutpoints for determining the level of an active smoker [21]. NNAL levels were measured using a liquid chromatography–tandem mass spectrometry at the University of California San Francisco; serum creatinine levels were used to correct for hydration status [22]. NNAL values were natural log transformed and analyzed as a continuous outcome. Values below the level of detection were coded as the limit of detection (0.25 pg/mg) divided by the square root of 2, consistent with NHANES analytic methods [23]. Adults with NNAL levels above the limit of detection were considered exposed to tobacco smoke.

### 2.2. Home Smoking Ban Categories for Household Pairs

Self-report of a home smoking ban was defined by confirming the home had a “no smoking rule” from the smoker and nonsmoker baseline surveys. Household pairs were grouped into three categories: (1) both members of the pair indicated a home smoking ban (home smoking ban), (2) only one member (either the smoker or nonsmoker) indicated a home smoking ban (inconsistent ban), and (3) both members of the pair indicated a no smoking ban (no home smoking ban). Among the 64 pairs with inconsistent reports, 22 smokers and 42 nonsmokers reported a ban while the other household member did not. Because of the small numbers and the fact that nonsmoker NNAL levels did not differ between these two subgroups, we combined pairs with inconsistent reports into a single category.

### 2.3. Covariates

Covariates examined included demographics, household characteristics, knowledge and attitudes about tobacco smoke exposure, and tobacco behavior. Demographics included age, sex, acculturation markers (years in the U.S. and English fluency: low fluency was defined as answering “not too well” or ”not at all” to “How well do you speak English?”), and education level (less than high school vs. at least a high school graduate). Household characteristics included the type of relationship between the respondents (e.g., married, relative, friend, caregiver), years lived together, presence of another smoker in the home, and presence of children in the home. Knowledge that tobacco smoke exposure caused disease or was harmful to health was assessed for 4 health conditions: lung cancer, heart disease, asthma, and children’s health; for this study, a variable was created for correctly identifying that tobacco smoke exposure was associated with all four conditions. Knowledge was also assessed for correctly identifying that ventilation does not eliminate exposure. Attitudes included perceived harmfulness of tobacco smoke exposure to the smoker or nonsmoker, confidence to maintain a smoke-free environment, and smokers’ ratings of nonsmoker household members providing helpful support for tobacco cessation. Behaviors included smoker quit attempt in the past year, smoker quit intent within the next 30 days, and smoking intensity (cigarettes consumed per day, smoker NNAL level), as higher smoking intensity may reflect degree of addiction [24]. Nonsmokers reported sources of tobacco smoke exposure in response to the question “In the past 2 months, where have you been exposed to smoke? (Circle all that apply)”. Responses were categorized as home, car, work (indoors or outdoors), outdoors, restaurant or bar, and other (other person’s home or car, casino, or other sources).

### 2.4. Statistical Analysis

Percentages and means were used to summarize the covariates by home smoking ban category. For categorical variables, *p*-values from chi-square tests (or Fisher’s Exact tests if cell was ≤5) were used to examine the association between each covariate and home smoking ban category. For continuous variables, *p*-values from ANOVA f-tests were used to test for differences in means between the three home smoking ban categories.

To examine the association between home smoking ban category and nonsmoker NNAL, we calculated geometric means and 95% confidence intervals. Unadjusted and adjusted linear regression models were conducted with natural log-transformed NNAL as the outcome. We examined each covariate for potential confounding of the association between home smoking ban category and nonsmoker log NNAL level by including each in the model one at a time. The final model included covariates that changed the effect estimate for home smoking ban status by more than 10%. A second model additionally adjusted for smoker log NNAL level to determine if smoking intensity may be an intermediate variable on the pathway between home smoking ban concordance and non-smoker NNAL level. Analysis was conducted using SAS version 9.4 (SAS institute, Cary NC).

## 3. Results

Table 1 shows the characteristics of the 202 Chinese American smoker and nonsmoker household pairs. Among the household pairs, the smokers were all male, and almost all of the nonsmokers were female (not shown). Most pairs were married (85.6%), with a mean of 20 years, and over three-quarters had children in the household; about 1 in 7 households had other smokers. The average age was 49.5 years for nonsmokers and 53.2 years for smokers. Nearly one-third of smokers and nonsmokers had less than a high school education. The average duration of U.S. residence was 8.9 years for nonsmokers and 10.8 for smokers. Nearly three-quarters of nonsmokers and smokers reported low English fluency.

Less than half of nonsmokers and slightly over half of smokers knew tobacco smoke exposure was associated with lung cancer, heart disease, asthma, and children’s health. Only about one-third of nonsmokers and smokers correctly identified that ventilation does not eliminate exposure. The majority of participants agreed that smoke was harmful to the smoker, but slightly fewer participants agreed that smoke was harmful to the nonsmoker; nonsmokers had overall higher percentages than smokers. The majority of participants agreed that they were confident to keep a smoke-free home environment, with smokers having overall higher percentages than nonsmokers. Nearly three-quarters of smokers agreed that their household nonsmoker was helpful in supporting quitting.

Among the household pairs, 53.9% reported a home smoking ban, 31.7% provided inconsistent ban reports, and 14.4% reported no ban (Table 1). Pairs with no home smoking bans had the highest proportion of nonsmokers with low English fluency (86.2%) and the lowest proportion of nonsmokers who were confident to maintain a smoke-free environment (58.6%). Pairs with no bans also had smokers with the highest mean number of cigarettes per day (13.5 cigarettes, SD = 8.9). Pairs with an inconsistent ban had a trend for lower percentages of those who were married than the other two pair groups. Pairs with inconsistent or no home smoking bans had a trend for higher percentages of other smokers in the house.

### 3.1. Nonsmoker Exposure to Secondhand Smoke

**Figure 1:**
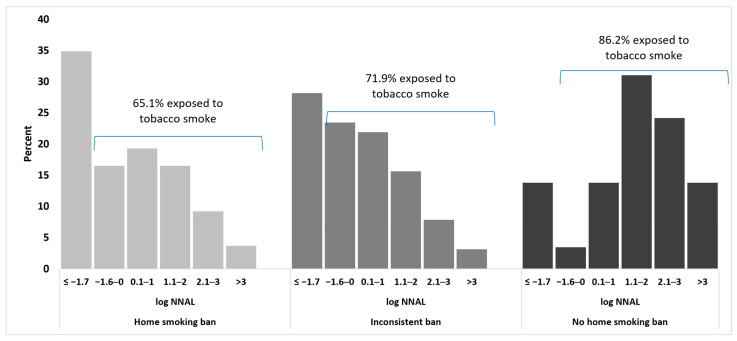
Distribution of nonsmoker NNAL (pg/mg) on the natural log scale by home smoking ban category.

Figure 1 shows the distribution of nonsmoker NNAL levels by home smoking ban category. The distributions of nonsmoker NNAL levels were shifted higher across home smoking ban categories, from a home smoking ban to an inconsistent ban to no home smoking bans. Accordingly, there were increasing proportions of nonsmokers with NNAL levels indicating tobacco smoke exposure: 66.1% home smoking ban, 71.9% inconsistent ban, and 86.2% no home smoking ban.

Among nonsmokers with detectable levels of NNAL, Table 2 describes self-reports of tobacco smoke exposure across various settings in the past two months. For nonsmokers with a home smoking ban, almost one-quarter reported tobacco smoke exposure in the home, the second highest source after outdoors. For nonsmokers with an inconsistent ban, less than one-third reported tobacco smoke exposure in the home, the second highest source after outdoors. For nonsmokers with no home smoking ban, over half reported tobacco smoke exposure in the home, the highest reported source. Nearly three-quarters (73.6%) of nonsmokers with a ban or inconsistent ban reported outdoor tobacco smoke exposure, compared with about one-third of nonsmokers with no home smoking ban. Between 10% and 20% of nonsmokers with a home smoking ban or inconsistent ban reported tobacco smoke exposure in settings other than inside the home and outdoors, while 20%–30% of nonsmokers with no home smoking ban generally reported tobacco smoke exposure in these other settings.

### 3.2. Multivariate Analysis

Table 3 shows the geometric mean of nonsmoker NNAL and the unadjusted or adjusted ratios by home smoking ban category. Nonsmokers without home smoking bans had higher geometric mean NNAL than those with inconsistent bans or a home smoking ban. Comparing across groups, the geometric mean NNAL for nonsmokers with no home smoking ban was four times higher than that of nonsmokers with a home smoking ban. A higher geometric mean NNAL was also seen with nonsmokers reporting low English fluency, smokers planning to quit beyond 6 months, and no children in the home. After adjusting for these covariates in Model 1, the geometric mean NNAL for nonsmokers without home smoking bans was over two times higher than that of nonsmokers with a home smoking ban; the geometric mean NNAL for nonsmokers with high English fluency was 50% lower than that of those with low English fluency. These findings did not differ after additionally adjusting for smoker NNAL level (Model 2). There was no statistically significant difference between nonsmokers with a home smoking ban versus inconsistent ban reports. Our final model explained 18% of the variation in tobacco smoke exposure.

## 4. Discussion

To our knowledge, this is the first study demonstrating that home smoking bans were associated with lower tobacco-specific nitrosamine biomarker levels of tobacco smoke exposure among Chinese American nonsmokers living with a current smoker. However, reports of home smoking bans did not equate to elimination of tobacco smoke exposure. Two-thirds of nonsmokers with a home smoking ban still had biomarker levels indicating tobacco smoke exposure, with some reporting continued tobacco smoke exposure within the home and many reporting outdoor tobacco smoke exposure. We also found that nearly one-third of household pairs had inconsistent reports of a home smoking ban, highlighting the importance of having reports from multiple household members for more accurate assessments.

Our study found that one-quarter of nonsmokers reported tobacco smoke exposure in the home despite having a home smoking ban, suggesting a need for more effective enforcement of home smoking bans. In our study, the proportion of Chinese American households with a home smoking ban (53.9%) was lower than other California households (67.9%) [25]. Encouraging more Chinese American households to establish a home smoking ban may foster a broader smoke-free social norm in the community. In a previous study of California Asian Americans and smoking bans at home or work, nonsmoker women with lower education levels reported more recent tobacco smoke exposure than their counterparts with higher education levels and may need to be empowered to assert and enforce their right for smoke-free environments [16]. California Asian American nonsmokers may need more support for enforcement, as almost two-thirds of them at highest risk of exposure asked someone not to smoke in the preceding year [26]. Such home smoking bans can also support California Asian Americans to quit smoking, especially for recent immigrants who have been in the U.S. for less than a decade [27].

Nonsmokers in our study had higher biomarker levels of tobacco smoke exposure overall than nonsmokers nationwide. While two-thirds to 86% of nonsmokers in our study had NNAL levels denoting tobacco smoke exposure, 41% of nonsmokers nationally had elevated NNAL levels, and NNAL was detected in 87.5% of those with substantial tobacco smoke exposure [28]. There is a need for stronger policy enforcement in the community setting for our study participants, as three-quarters of those with a home smoking ban reported the outdoors as the leading source of exposure. Despite California’s strong smoke-free policies, over half of Californians still reported tobacco smoke exposure in the past 2 weeks [29]. Although San Francisco has a comprehensive smoke-free outdoor air policy that prohibits smoking in outdoor public transit waiting areas, outdoor dining areas, and outdoor parks [30], it is an urban environment where nonsmokers may be exposed to smoke on the sidewalk or other outdoor areas. While not specifically asked in the study, multiunit housing in urban areas may be another important source of exposure, as smoke travels through ventilation and the built environment [4].

Our finding that nearly one-third of household pairs had inconsistent ban reports underscores that relying on a single respondent may result in the misreporting of home smoking bans. Two-thirds of nonsmokers in these pairs thought they had a home smoking ban while their household smokers did not. Our results of inconsistent reports are similar to a previous study by Ding et al., where 19% of 386 parent/child pairs had discordant reports of home smoking bans [15]. In that study, over one-third of the parents and nearly half of the children reported at least an occasional ban violation even when both agreed on having a home smoking ban. Our study found higher proportions of nonsmokers exposed to smoke in households with inconsistent bans when compared with households with home smoking bans, but there was no difference between the geometric mean NNAL levels of nonsmokers. Of note, both groups had nearly one-quarter to one-third of nonsmokers reporting tobacco smoke exposure in the home in the past couple of months. This could reflect intermittent lapses in ban enforcement, different interpretations of a “no smoking rule”, thirdhand tobacco smoke exposure, or significant tobacco smoke exposure from other sources outside the respondents’ living spaces (e.g., shared areas, hallways, or balconies in multiunit housing) [16,25].

Improving knowledge about tobacco smoke exposure and strategies to intervene are needed for Chinese American immigrant households, especially those with low English fluency. Most smokers and nonsmokers in this study believed that smoke was harmful but had lower levels of knowledge about secondhand smoke health harms and ventilation. Most smokers and nonsmokers in the study also reported confidence in keeping a smoke-free environment but nearly one-quarter to one-half of nonsmokers reported tobacco smoke exposure in the home. These contradictions may reflect limited understanding about tobacco smoke exposure for this population. In previous focus groups of similar Chinese American household pairs [31], participants focused more on the irritating odor than the health harms of exposure and had inaccurate beliefs about the harms of smoking and cessation. Still, smokers in the focus groups recognized that social acceptability and environmental regulation was a different social norm and that quitting could improve family harmony.

The Chinese American household pairs in this study subsequently participated in an educational intervention about smoke-free living and cessation support resources [17]; the latter including the nationwide Asian Smokers Quitline available in Chinese, Korean, and Vietnamese languages. After the intervention [17], nonsmokers had significantly reduced NNAL levels from the baseline levels reported here. Household pairs interviewed after the trial reported that learning about the health harms of tobacco smoke exposure and communication strategies helped to improve support for smoke-free living and household relationships [32]. This underscores the important role health professionals can play to screen nonsmokers about tobacco smoke exposure at home, ask about the presence of children or other smokers in the household, counsel about establishing and enforcing a home smoking ban, and encourage household smokers to quit [4].

Tobacco biomarkers demonstrating tobacco smoke exposure may help people understand their health risk better, but several considerations are needed. The assay used to measure the long-term tobacco biomarker NNAL in this study is not yet widely available in commercial laboratories. An alternative biomarker, cotinine, is a nicotine metabolite that is more widely available, although not necessarily available at high-sensitivity levels to detect tobacco smoke exposure, and NNAL may be slightly more specific [20]. In the parent study intervention [17], Chinese American household pairs in the intervention group were provided one biomarker feedback report using NNAL for each smoker or nonsmoker. Household pairs interviewed after the trial reported that biomarker feedback reports were useful and important but had short-term effects for motivating the smoker to quit [32].

### Limitations

A limitation of this study is that the parent study was not powered to detect differences in NNAL levels by home smoking ban category, although the percentages of nonsmokers with detectable NNAL levels did have a statistical trend in the hypothesized direction by home smoking ban category. As the study was conducted in an urban area in northern California, results may not be generalizable to Chinese Americans residing in regions where tobacco control is a lower priority and where there are fewer public health or policy interventions. Generalizability may also be limited by participant recruitment through community organizations, health care facilities, and health fairs; participants may have been more concerned about their health compared to nonparticipants. We specifically recruited households with a current cigarette smoker but did not determine if other tobacco products were used or exempted from any home smoking bans. Finally, as this study is cross-sectional, we can only measure associations between home smoking bans and decreased tobacco smoke exposure; it is possible that households with lower exposure to smoke are more likely to adopt home smoking bans.

## 5. Conclusions

This study demonstrates the importance of home smoking bans in reducing nonsmoker tobacco smoke exposure among Chinese American household pairs. However, there is still a need to support this population from a health equity standpoint, as nonsmokers had tobacco smoke exposure and reported outdoor exposure at percentages above those of the general population. Targeted health education and outreach may address the lower levels of knowledge about smoke harms and ventilation issues across all participants and assist the large proportion of nonsmokers with a home smoking ban who still reported home exposure. Tobacco biomarkers may also help with monitoring exposure and accountability for smoke-free policy compliance. Future research should consider how findings from Chinese American immigrant households may extend internationally to their counterparts in China, where large scale smoke-free public policies have helped decrease smoking prevalence and tobacco smoke exposure [33].

## Figures and Tables

**Table 1:** Characteristics of Chinese American nonsmoker and smoker household pairs by home smoking ban category.

Characteristics	Total	Home Smoking Ban Category			
		Ban	Inconsistent	No Ban	*p*-Value
Participant pairs, *n* (%)	202	109 (53.9%)	64 (31.7%)	29 (14.4%)	
**Household pairs**					
Married, *n* (%)	173 (85.6%)	98 (89.9%)	49 (76.6%)	26 (89.7%)	0.05
Years pairs lived together, mean (std)	20.4 (14.5)	18.3 (14.1)	21.8 (13.9)	25.1 (16.2)	0.06
Children in household (*n* = 143), *n* (%)	109 (76.2%)	60 (73.2%)	33 (82.5%)	16 (76.2%)	0.58
Other smoker (besides partner) in the house, *n* (%)	28 (14.1%)	9 (8.4%)	13 (20.6%)	6 (20.7%)	0.04
**Nonsmoker**					
Age, mean (std)	49.5 (13.0)	48.8 (12.7)	49.2 (13.7)	52.8 (12.6)	0.34
Less than high school education, *n* (%)	64 (31.8%)	34 (31.5%)	17 (26.6%)	13 (44.8%)	0.21
Years lived in the U.S., mean (std)	8.9 (8.0)	7.8 (7.2)	10.0 (7.9)	10.7 (10.3)	0.10
Low English fluency: “not too well/not at all”, *n* (%)	143 (71.1%)	68 (63.0%)	50 (78.1%)	25 (86.2%)	0.02
Knowledge, *n* (%)					
Tobacco smoke exposure harms: 4 health conditions	95 (47.3%)	54 (50.0%)	32 (50.0%)	9 (31.0%)	0.57
Ventilation does not eliminate exposure	76 (37.8%)	40 (37.0%)	24 (37.5%)	12 (41.4%)	0.91
Attitudes, *n* (%)					
Smoke is harmful to smoker	179(89.5%)	99 (92.5%)	55 (85.9%)	25 (86.2%)	0.28
Smoke is harmful to nonsmoker	160 (80.0%)	88 (82.2%)	49 (76.6%)	23 (79.3%)	0.66
Confident to keep a smoke-free home	149 (74.5%)	87 (81.3%)	45 (70.3%)	17 (58.6%)	0.03
**Smoker**					
Age, mean (std)	53.2 (14.3)	52.5 (14.2)	52.7 (14.4)	57.2 (14.5)	0.28
Less than high school education, *n* (%)	65 (32.3%)	37 (33.9%)	18 (28.6%)	10 (34.5%)	0.74
Years lived in the U.S., mean (std)	10.8 (9.6)	10.0 (9.6)	11.7 (9.0)	12.0 (10.6)	0.42
Low English fluency: “not too well/not at all”, *n* (%)	153 (76.1%)	79 (72.5%)	51 (81.0%)	23 (79.3%)	0.41
Knowledge, *n* (%)					
Tobacco smoke exposure harms: 4 health conditions	117 (58.2%)	71 (65.1%)	33 (52.4%)	13 (44.8%)	0.20
Ventilation does not eliminate exposure	64 (32.3%)	41 (37.6%)	13 (21.3%)	10 (35.7%)	0.09
Attitudes, *n* (%)					
Smoke is harmful to smoker	153 (76.1%)	81 (74.3%)	52 (82.5%)	20 (69.0%)	0.30
Smoke is harmful to nonsmoker	141 (70.2%)	75 (68.8%)	46 (73.0%)	20 (69.0%)	0.84
Confident to keep a smoke-free home	170 (84.6%)	96 (88.1%)	53 (84.1%)	21 (72.4%)	0.12
Non-smoker helpful in quitting	144 (72.4%)	81 (75.0%)	44 (69.8%)	19 (67.9%)	0.65
Tobacco behavior, *n* (%)					
Quit attempt in past year	108 (53.5%)	64 (58.7%)	33 (51.6%)	11 (37.9%)	0.13
Plan to quit in the next 30 days	56 (28.0%)	35 (32.1%)	14 (21.9%)	7 (25.9%)	0.25
Cigarettes smoked per day, mean (std)	10.2 (6.6)	10.1 (6.3)	8.9 (5.2)	13.5 (8.9)	0.01
NNAL, geometric mean in pg/mg, (std)	47.3 (7.1)	46.3 (7.3)	41.5 (6.7)	67.5 (7.5)	0.54

NNAL: 4-(methylnitrosamino)-1-(3-pyridyl)-1-butanol, std: standard deviation.

**Table 2:** Self-reported past 2 month tobacco smoke exposure settings among nonsmokers with detectable NNAL levels (*n* = 143).

Setting	Home Smoking Ban Category					
	Ban (*n* = 72)		Inconsistent Ban (*n* = 46)		No Ban (*n* = 25)	
	*n*	Percent	*n*	Percent	*n*	Percent
Home	17	23.6%	14	30.4%	14	56.0%
Car	9	12.5%	7	15.2%	2	8.0%
Work	11	15.3%	4	8.7%	7	28.0%
Outdoors	53	73.6%	35	76.1%	9	36.0%
Restaurant or bar	10	13.9%	8	17.4%	6	24.0%
Other	13	18.1%	8	17.4%	5	20.0%

Note: Participants can report more than one category of tobacco smoke exposure. “Other” includes other person’s home or car, casino, and other sources.

**Table 3:** Nonsmoker NNAL geometric mean and ratio of geometric means by home smoking ban category.

Home Smoking Ban Category and Covariates	Geometric Mean (95% CI) (pg/mg)	Unadjusted Ratio of Geometric Means (95% CI)	*p*-Value	Model 1 Adjusted Ratio of Geometric Means (95% CI)	*p*-Value	Model 2 Adjusted Ratio of Geometric Means (95% CI)	*p*-Value
Total	1.24 (0.98, 1.56)						
Home smoking ban							
Ban	0.97 (0.71, 1.33)	Reference		Reference		Reference	
Inconsistent	1.06 (0.73, 1.54)	1.09 (0.66, 1.81)	0.73	0.82 (0.43, 1.55)	0.54	0.96 (0.52, 1.77)	0.90
No ban	4.23 (2.18, 8.22)	4.35 (2.23, 8.50)	< 0.001	2.61 (1.11, 6.09)	0.03	2.65 (1.18, 5.94)	0.02
Smoker log NNAL		1.38 (1.24, 1.55)	< 0.0001			1.34 (1.16, 1.54)	< 0.0001
Nonsmoker English fluency							
Low	1.53 (1.16, 2.05)	Reference		Reference		Reference	
High	0.74 (0.50, 1.10)	0.48 (0.29, 0.81)	0.006	0.50 (0.27, 0.94)	0.03	0.54 (0.30, 0.98)	0.04
Smoker planning to quit							
Next 6 months	0.91 (0.67, 1.24)	0.53 (0.33, 0.84)	0.007	0.67 (0.39, 1.16)	0.15	0.80 (0.47, 1.35)	0.40
>6 months	1.72 (1.21, 2.45)	Reference		Reference		Reference	
Children in home							
Yes	1.22 (0.89, 1.67)	Reference		Reference		Reference	
No	1.63 (0.88, 3.04)	1.34 (0.70, 2.58)	0.38	1.03 (0.54, 1.97)	0.93	1.14 (0.61, 2.11)	0.68
Adjusted R squared		0.08		0.08		0.18	

Model 1 adjusted for nonsmoker speaks English “not too well/not at all”, child in the home, and smoker planning to quit in next 6 months. Model 2 additionally adjusted for smoker log NNAL.

## Data Availability

The data presented in this study are available upon request from the corresponding author.

## Trial Registration

The trial is registered at clinicaltrials.gov (National Clinical Trials identifier NCT01592682).
